# CTPA, DECT, MRI, V/Q Scan, and SPECT/CT V/Q for the noninvasive diagnosis of chronic thromboembolic pulmonary hypertension: A protocol for systemic review and network meta-analysis of diagnostic test accuracy

**DOI:** 10.1097/MD.0000000000016787

**Published:** 2019-08-23

**Authors:** Shuanglan Xu, Jiao Yang, Yun Zhu, Shuangyan Xu, Jie Liu, Yishu Deng, Li Wei, Mei Yang, Xiaoxian Huang, Bing Cao, Chunfang Zhang, Fangyun Zhao, Xing Liu, Xiqian Xing, Zhongming Li

**Affiliations:** a Department of Respiratory Medicine, The Fourth Affiliated Hospital of Kunming Medical University, The Second People's Hospital of Yunnan Province; b First Department of Respiratory Medicine, The First Affiliated Hospital of Kunming Medical University; c The People's Hospital of Yuxi City, The 6th Affiliated Hospital of Kunming Medical University, Yuxi; d Department of Dermatology, The Second Affiliated Hospital of Kunming Medical University; e Department of Pharmacy, Yan’an Hospital Affiliated to Kunming Medical University; f Department of Anatomy, Basic Medical Sciences of Kunming Medical University, Kunming, Yunnan, China.

**Keywords:** chronic thromboembolic pulmonary hypertension, diagnostic, network meta-analysis, noninvasive diagnosis, protocol

## Abstract

**Background:**

To determine the diagnostic accuracy of techniques with chronic thromboembolic pulmonary hypertension (CTEPH) patients via a protocol for systemic review and network meta-analysis.

**Methods:**

We will search PubMed, EMBASE, Web of Science, and Google Scholar from inception to October 1, 2018. The reference lists of the retrieved articles are also consulted. Quality Assessment of Diagnostic Accuracy Studies 2 (QUADAS-2) will be used to assess the risk of bias in each study. The direct meta-analyses, network meta-analyses, and ranking of competing diagnostic tests will be used by STATA 12.0 and WINBUGS 1.4. Heterogeneity and inconsistency are assessed.

**Results:**

This study is ongoing, will be submitted to a peer-reviewed journal publication once completed.

**Conclusion:**

This study will provide a comprehensive evidence summary of diagnostic test accuracy in detecting the CTEPH, and can help patients and clinicians to select appropriate or best diagnostic test.

**Ethics and Communication:**

No ethical approval and patient consent are required, because it is based on published researches.

**PROSPERO registration number:**

CRD42019121279.

## 1 Introduction

Pulmonary hypertension (PH) is defined as an elevated mean pulmonary arterial pressure (mPAP) ≥25 mm Hg and pulmonary artery wedge pressure ≤15 mm Hg at rest as measured by invasive right heart catheterization (RHC).^[1]^ Chronic thromboembolic pulmonary hypertension (CTEPH) is the clinical classification of PH. It is a progressive pulmonary vascular disease with significant morbidity and mortality.^[2]^ However, if went through a successful surgical treatment or catheter-based intervention, substantial improvement can be obtained in right ventricular function, exercise capacity, gas exchange, and quality of life.^[3]^ Unfortunately, the patient cannot use this treatment at the time of diagnosis. This might be partly the signs and symptoms are not specific or even undetected in the early stage of, CTEPH remains largely underdiagnosed.

**Figure 1:**
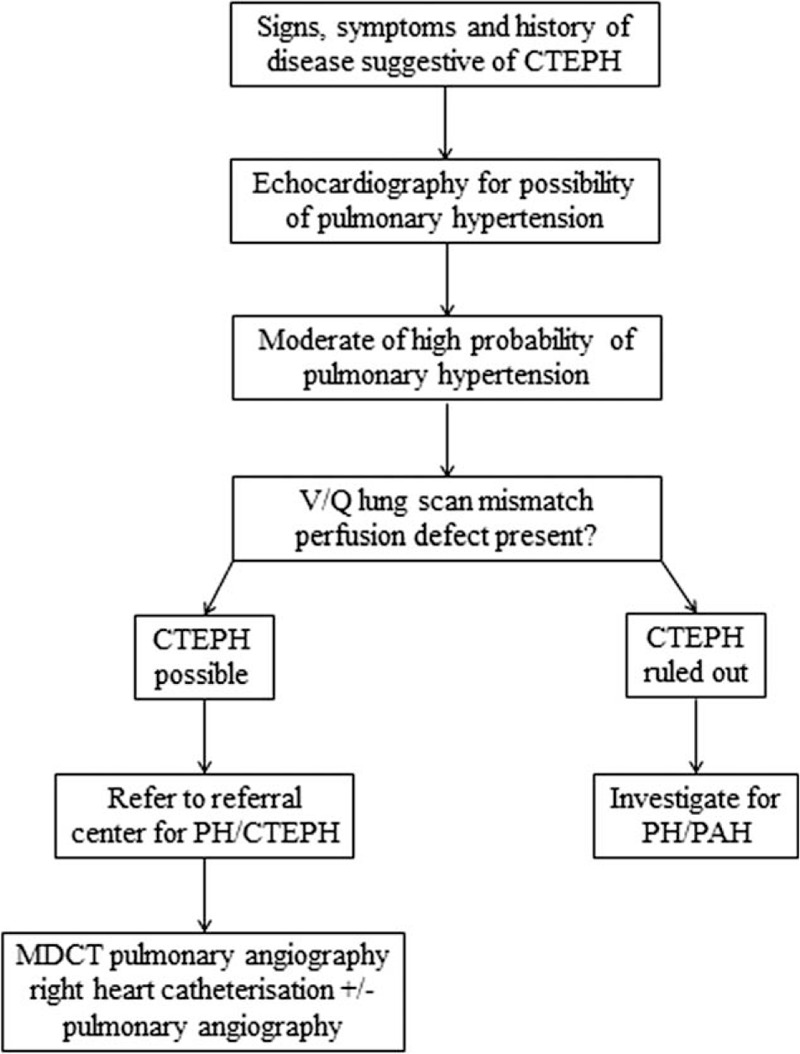
Diagnosis flow of CTEPH. CTEPH = chronic thromboembolic pulmonary hypertension.

To date, conventional pulmonary angiography remains the gold standard for confirming the diagnosis of CTEPH. However, with the development of noninvasive diagnosis technique, this invasive technique is mandatory no more to confirm the diagnosis. The guidelines of European Respiratory Society (ERS) and European Society of Cardiology (ESC) published in 2015 can provide an algorithm to diagnose CTEPH.^[4]^ And diagnosis flow of CTEPH based on this guideline show that such test can proved useful diagnose of CTEPH (Fig. 1), including echocardiography, ventilation perfusion scan (V/Q Scan), computed tomography pulmonary angiography (CTPA), RHC and pulmonary angiography (PA). Dual energy computed tomography (DECT) and single photon emission computed tomography SPECT/CT V/Q scanning as newer modalities, which have emerged in the past decade, and used to the diagnosis of CTEPH now. However, it is still unclear which individual test or combined test is the best for the noninvasive diagnosing CTEPH based on currently available studies. Thus, it is important to find specific test for this type of PH.

We know that network analysis is widely used to evaluate the effectiveness and safety of different drugs. No network meta-analysis to rank the diagnostic test accuracy for CTEPH now. Therefore, we aimed to evaluate and compare the diagnostic accuracy of techniques for CTEPH using network meta-analysis method, and to rank these test using superiority index.

## 2 Methods

### 2.1 Design and registration

We will conduct a network meta-analysis of diagnostic test accuracy. The study protocol has been registered on the international prospective register of systematic review (PROSPERO), the registration number was CRD42019121279. We will follow the Preferred Reporting Items for Systematic Reviews and Meta-Analyses (PRISMA)^[5]^ statements for reporting our systematic review.

### 2.2 Information sources

We searched PubMed, EMBASE, Web of Science, and Google Scholar from inception to October 1, 2018 to identify relevant eligible systematic reviews and meta-analyses.

### 2.3 Search strategy

The search strategy was designed and conducted by an experienced medical librarian with input from study investigators. The Medical Subject Heading (MeSH) terms and free keywords used to identify article. We combined search terms for applied technique (CTPA, dual-energy computed tomography [DECT], MRI, V/Q scan, or SPECT/CT V/Q) and disease (chronic thromboembolic pulmonary hypertension, CTEPH).

### 2.4 Inclusion and exclusion criteria

The inclusion criteria were as follows: a cohort study or case-control study; a suspected or confirmed diagnosis of CTEPH based on World Health Organization (WHO) Evian/Venice classification; determination of tests including CTPA, DECT, MRI, V/Q Scan, or SPECT/CT V/Q; an evaluation of the association between technique tests and the diagnosis of CTEPH patients; and the presence of sufficient data including true positive (TP), false positive (FP), true negative (TN), and false negative (FN) to calculate the sensitivity (SEN), specificity (SPE), and diagnostic odds ratios (DOR). Studies were excluded based on the following criteria: a review, abstract, case report, or letter; use of animals; and duplicate report. If the data were duplicated or the same population was used in >1 study, we chose the most recent or complete study. The published language was not limited.

### 2.5 Study selection

**Figure 2:**
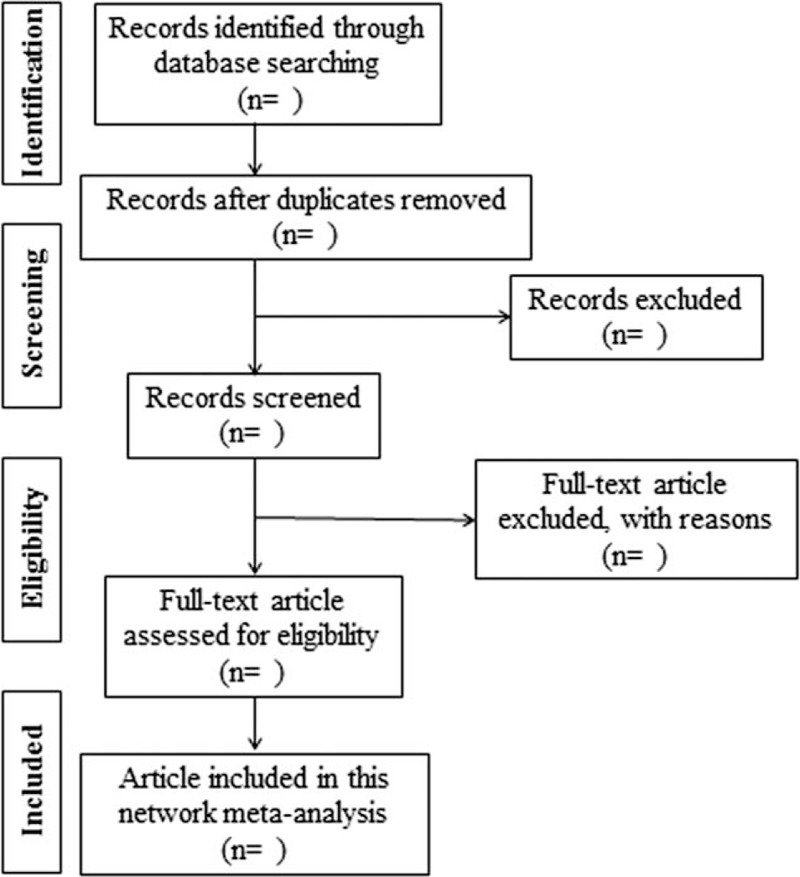
Flow chart of searching and screening studies.

We will import initial search records form databases into ENDNOTE X8 (Clarivate Analytics, 2017) literature management software. Two independent investigators (XXQ and XSL) examined titles and abstracts of all search results. After one investigator regarded a study as potentially eligible, we read the full article and resolved disagreements by discussion. The flow chart of searching and screening studies is showed at the Fig. 2.

### 2.6 Data extraction

According to the aforementioned selection criteria, data extraction was performed by one investigator (XSL) using a standardized data abstraction form, and verified by a second investigator (XXQ). Any disagreements were resolved by discussion. The following information was extracted from the eligible studies: studies characteristics (i.e., the name of the first author, publication year, study period, country, study type, gold standard, index test), patients characteristics (i.e., sample number, sex of patients, mean age or age range, non-invasive method, cut-off level, risk factors of CTEPH), and outcomes (TP, FP, FN, TN, SEN, SPE, DOR). If available, data were recorded on patient basis (i.e., past history about pulmonary thromboembolism or deep vein thrombosis) and vessel basis (i.e., main + lobar pulmonary arteries and segmental arteries).

### 2.7 Quality evaluation

The Quality Assessment of Diagnostic Accuracy Studies 2 (QUADAS-2)^[6]^ will used for methodological quality evaluation for each study, and has 2 parameters: risk of bias and concerns regarding applicability. High-quality, low-biased literature will be screened for this work. Two reviewers will independently assess, and conflicts will be resolved by discussion.

### 2.8 Statistical analysis

#### 2.8.1 Direct meta-analyses

We will perform pairwise meta-analyses for all diagnostic tests comparisons, the *Q*-test and *I*^2^ test will be used to assess heterogeneity among the studies.^[7]^ We also calculated the *P* values of the *Q*-test, which represent heterogeneity; values of *P* ≤ .10 or *I*^2^ ≥ 50% indicated heterogeneity among studies, and a D-L random random-effect model was used. Otherwise, the M-H fixed-effects model was applied. Data analyses will be performed using STATA statistical software version 12.0 (STATA Corp. LLC, College Station, TX).

#### 2.8.2 Network meta-analyses

**Figure 3:**
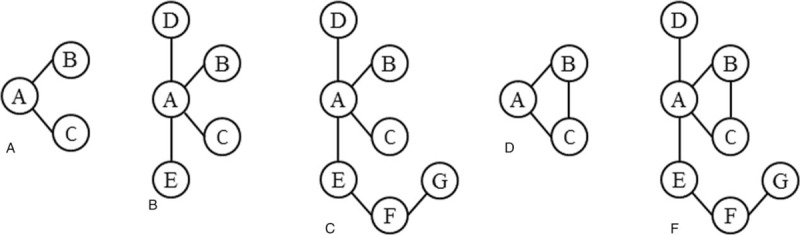
Type of network plot.

A network is including head-by-head comparisons and indirect comparisons. Type of network plot have showed (Fig. 3), a node represents a test, the thickness of the lines between the nodes is proportional to the direct comparisons between tests, and the size of the nodes is proportional to the number of studies evaluating a test. We make a hypothesis V/Q scan as fundamental reference test, the relative diagnostic outcomes between different tests will calculated, including relative SEN, relative SPE, and relative DOR.^[8]^ Data analyses will be conducted using STATA statistical software version 12.0 (STATA Corp. LLC, College Station, TX) and Bayesian Inference Using Gibbs Sampling (WINBUGS 1.4, MRC Biostatistics Unit, England).

#### 2.8.3 Ranking of competing diagnostic tests

We will rank diagnostic test in order of their accuracy using the surface under the cumulative ranking (SUCRA).

#### 2.8.4 Heterogeneity and consistency test

The strategies employed to explore the origin of pairwise meta-analysis, also can be used in network analysis to tackle inconsistency.^[9]^ Differences between direct and indirect evidence were assessed using tests of model consistency by including trial design as an additional covariate in the model. Whether the heterogeneity or inconsistency among the studies will be detected, subgroup analysis, sensitivity analysis or meta-regression model will be performed to explore the possible sources of heterogeneity or inconsistency.

Subgroup analysis of variables is including sample size, severity of CTEPH, diagnostic duration. A sensitivity analysis used to evaluate the robustness of our analysis by recalculating the pooled results from the primary analyses after excluding one study per iteration.

#### 2.8.5 Publication bias

There are >10 studies, the Deek funnel plot will be applied to evaluate the extent of potential publication bias.^[10]^ Otherwise, the Begg rank correlation test and Egger linear regression test will be applied.

#### 2.8.6 Patient and public involvement

Not involved.

## 3 Discussion

Before on the most previous studies, we found that this will be the first diagnostic network meta-analysis comprehensively comparing different diagnostic accuracy of techniques for CTEPH.

As we all known, many tests can be applied as diagnostic tools for CTEPH, inclusive of invasive and non-invasive. There is the first meta-analysis carried out to estimate the role of CT in detection of CTEPH, it indicated a moderate sensitivity and a high specificity of CT for the assessment of CTEPH on patient basis, therefore CT can be used as a good tool for the diagnosis of CTEPH.^[11]^ With the development of technology, DECT has introduced into clinical practice in 2006, and it is used maturely to detect physiological gradients of lung perfusion and subtle perfusion defects.^[12]^ DECT is more accurate than angiography at identifying the segmental location of abnormalities.^[13]^ Recent studies have shown that the diagnostic value for DECT was consistent with V/Q Scan excellently.^[14]^

Tunaria's group showed V/Q scan have the sensitivity (96–97.4% vs 51%) and the specificity (90–95% vs 99%) compared with CTPA. So, they believe their results demonstrate that V/Q scan has a higher sensitivity than CTPA in detecting CTEPH.^[15]^ Compared with CTPA, V/Q scans with less radiation have 2 advantages, avoid complications secondary to administration of intravenous contrast, other one is more cost effective. Furthermore, 2015 ESC/ERS Guidelines showed the V/Q scan as the fastest imaging method to diagnose CTEPH, gives 96 to 97% sensitivity and 90 to 97% specificity.^[4]^ However, V/Q scan is not specific for CTEPH, false positive results can be seen in pathologies such as fibrosing mediastinitis,^[16]^ pulmonary artery sarcoma.^[17]^ What's more, several studies have performed that the superiority of SPECT/CT V/Q imaging tool over planar techniques in the assessment of pulmonary thromboembolic disease.^[18]^

Combining digital subtraction angiography (DSA) and right heart catheterization, it can provide the choice of diagnosing CTEPH and assess the eligibility of surgery.^[19]^ But DSA not be widely used yet, because it's invasive and expensive.^[20]^ Contrast-enhanced MRI has high sensitivity and specificity for diagnosing CTEPH compared with CT pulmonary angiography.^[21]^ However, uncertainty still persists about the performance of MRI. But now, it is still unclear which test will be favorable among those tests for the patient with CTEPH. Hence, this study will provide a comprehensive evidence summary of diagnostic test accuracy in detecting the CTEPH, in order to help clinicians and patients.

## Author contributions

**Conceptualization:** Xiqian Xing, Shuanglan Xu, Jiao Yang, Yun Zhu, Zhongming Li.

**Data curation:** Xiqian Xing, Shuanglan Xu, Jiao Yang, Yun Zhu.

**Formal analysis:** Xiqian Xing, Shuanglan Xu, Jiao Yang, Yun Zhu, Zhongming Li.

**Funding acquisition:** Xiqian Xing.

**Investigation:** Yishu Deng, Li Wei, Mei Yang, Xiaoxian Huang.

**Methodology:** Yishu Deng, Li Wei, Mei Yang, Xiaoxian Huang.

**Project administration:** Shuangyan Xu, Jie Liu, Bing Cao.

**Resources:** Shuangyan Xu, Jie Liu, Bing Cao.

**Software:** Chunfang Zhang, Fangyun Zhao, Xing Liu.

**Supervision:** Chunfang Zhang, Fangyun Zhao, Xing Liu.

**Validation:** Xiqian Xing, Zhongming Li.

**Visualization:** Xiqian Xing, Zhongming Li.

**Writing – original draft:** Shuanglan Xu.

**Writing – review & editing:** Jiao Yang, Yun Zhu.

Xiqian Xing orcid: 0000-0002-5359-1778.
